# Patient participation in electronic nursing documentation: an interview study among community nurses

**DOI:** 10.1186/s12912-021-00590-7

**Published:** 2021-05-01

**Authors:** Kim De Groot, Elisah B. Sneep, Wolter Paans, Anneke L. Francke

**Affiliations:** 1 Netherlands Institute for Health Services Research (Nivel), PO Box 1568, 3513 CR Utrecht, The Netherlands; 2 Nursing Science, Programme in Clinical Health Sciences, University Medical Centre Utrecht, Utrecht University, PO Box 85500, 3508 GA Utrecht, The Netherlands; 3 Research Group Nursing Diagnostics, School of Nursing, Hanze University of Applied Sciences, Petrus Driessenstraat 3, 9714 CA Groningen, The Netherlands; 4 Department of Critical Care, University Medical Centre Groningen, PO Box 30.001, 9700 RB Groningen, The Netherlands; 5 Department of Public and Occupational Health, Amsterdam Public Health Research Institute, Amsterdam University Medical Centre, Vrije Universiteit Amsterdam, Van der Boechorststraat 7, 1081 BT Amsterdam, The Netherlands

**Keywords:** Patient participation[MeSH], Nursing documentation, Electronic health record[MeSH], Home care

## Abstract

**Background:**

Patient participation in nursing documentation has several benefits like including patients’ personal wishes in tailor-made care plans and facilitating shared decision-making. However, the rise of electronic health records may not automatically lead to greater patient participation in nursing documentation. This study aims to gain insight into community nurses’ experiences regarding patient participation in electronic nursing documentation, and to explore the challenges nurses face and the strategies they use for dealing with challenges regarding patient participation in electronic nursing documentation.

**Methods:**

A qualitative descriptive design was used, based on the principles of reflexive thematic analysis. Nineteen community nurses working in home care and using electronic health records were recruited using purposive sampling. Interviews guided by an interview guide were conducted face-to-face or by phone in 2019. The interviews were inductively analysed in an iterative process of data collection–data analysis–more data collection until data saturation was achieved. The steps of thematic analysis were followed, namely familiarization with data, generating initial codes, searching for themes, reviewing themes, defining and naming themes, and reporting.

**Results:**

Community nurses believed patient participation in nursing documentation has to be tailored to each patient. Actual participation depended on the phase of the nursing process that was being documented and was facilitated by patients’ trust in the accuracy of the documentation. Nurses came across challenges in three domains: those related to electronic health records (i.e. technical problems), to work (e.g. time pressure) and to the patients (e.g. the medical condition). Because of these challenges, nurses frequently did the documentation outside the patient’s home. Nurses still tried to achieve patient participation by verbally discussing patients’ views on the nursing care provided and then documenting those views at a later moment.

**Conclusions:**

Although community nurses consider patient participation in electronic nursing documentation important, they perceive various challenges relating to electronic health records, work and the patients to realize patient participation. In dealing with these challenges, nurses often fall back on verbal communication about the documentation. These insights can help nurses and policy makers improve electronic health records and develop efficient strategies for improving patient participation in electronic nursing documentation.

## Background

Accurate and complete nursing documentation is known to promote the quality and continuity of care [1–3]. Nursing documentation is defined as: ‘the process of documenting nursing information about nursing care in health records’ [4]. Documentation needs to be efficient and logically arranged, and therefore structured according to the phases of the nursing process, namely assessment, nursing diagnosis, care planning, implementation of interventions, and evaluation of care or — if relevant — handover of care [1, 5, 6].

According to Jefferies et al., another criterion for nursing documentation is that it should include the patients’ views on their condition and their response to nursing care [7]. When nursing documentation is completed in consultation with patients and includes their personal wishes, this is a form of patient participation.

Patient participation in nursing documentation is not only a form of patient participation in its own right, but it also promotes patient participation in other aspects of nursing care. A study by Vestala and Frisman showed that when nurses discuss matters with patients that patients perceive to be important to have documented, patients are better able to express their thoughts about the care directly [8]. This can therefore facilitate shared decision-making about the nursing care. Moreover, this decision process can, in turn, result in better tailored care plans, in which the personal wishes of patients are addressed.

On top of that, patient participation in nursing documentation can also improve the accuracy of the documentation. Several studies have reported inconsistencies between the content of nursing documentation and the nursing care provided, showing that further improvement in the accuracy of the documentation is urgently needed [9–11].

The aforementioned benefits from patient participation are also reflected in laws and regulations. Today, legal requirements in many Western countries (e.g. Canada, Norway, the USA and the Netherlands) support patient participation in nursing documentation and state that patients must have access to health care professionals’ documentation [12–15]. Moreover, Dutch legislation states that patients’ access to their health records should be arranged electronically [16]. Furthermore, this legislation states that patients have the right to supplement, correct and delete information in the health records [15].

Additionally, several professional quality standards and guidelines refer to the importance of patient participation in nursing documentation [17, 18]. For instance, the Dutch national guideline for nursing documentation recommends that all phases of the nursing process should be documented in consultation with the patient [19].

The rise of electronic patient portals could in theory make it easier to achieve patient participation in nursing documentation [20–22]. Electronic patient portals are applications that allow patients to electronically access health records managed by a care organization. With these applications, patients can access their health records independently of their health care professionals and at their own preferred time. Electronic patient portals are being used across various health care sectors, but in the Netherlands the home care sector in particular is leading the way in the use of such portals. A recent survey among Dutch nursing staff showed that 81 % of community nurses said that their organization worked with an electronic patient portal [22].

However, the rise of electronic health records and electronic patient portals may not automatically lead to more patient participation in nursing documentation. In the past, the paper-based health records of home care organizations remained in the patient’s home and were in principle easily accessible for the patient. Using electronic patient portals, however, requires some digital skills to access the electronic health records, which can be challenging for some patients [23–25]. One Dutch study, consisting of a survey among nursing staff and a focus group with patients and family caregivers, indicated that some patients feel they have limited participation in nursing documentation [26]. Until now, however, there has hardly been any empirical research addressing community nurses’ experiences of patient participation in nursing documentation.

The objectives of the present study were therefore (a) to gain insight into community nurses’ experiences regarding patient participation in electronic nursing documentation; (b) to explore what challenges community nurses face, and what strategies they use to deal with the challenges regarding patient participation in electronic nursing documentation.

## Methods

### Design

A qualitative descriptive design was used, following the steps of reflexive, inductive thematic analysis [27, 28]. Thematic analysis aims to identify meaningful themes across a dataset [27], in this case transcripts of semi-structured interviews.

### Participants and setting

Nurses were eligible to participate in this study if they met all of the following inclusion criteria:

1. Being a registered nurse with a bachelor’s degree or a secondary vocational qualification in nursing;
2. Currently working in home care;
3. Using electronic health records.

Dutch community nurses either have a secondary vocational qualification (after a four-year nursing training programme at a regional centre for secondary vocational education) or a bachelor’s degree (after a four-year nursing training programme at a university of applied sciences). We included nurses from both educational levels.

Participants were recruited through the nationwide network of the Dutch College of Community Nurses (Nederlands Wijkverpleegkundigen Genootschap) and the professional network of two of the authors (ES and KdG). Additionally, snowball sampling was used by asking participants whether they knew other community nurses who would like to participate in the present study. Furthermore, purposive sampling was applied so that there would be variation between participants in terms of:

1. The standardized terminology used in the electronic health records, taking into account the fact that Dutch home care providers are obligated to implement standardized terminologies in their health records [29], and that the Omaha System — a standardized terminology originally developed for the public domain — is the most common terminology used in Dutch home care [4, 30];
2. The software package for the electronic health records, taking into account that the software package supplied by the developer Nedap is the most common package used in home care in the Netherlands;
3. Working experience as a nurse (in years).

These characteristics were expected to influence community nurses’ experiences with patient participation in electronic nursing documentation.

Participants were recruited for interviews until data saturation was reached. No new information relevant to the objectives of the study was obtained in the 17th interview. Two more interviews were held to confirm data saturation, giving 19 interviews in total.

### Data collection

The 19 interviews were conducted between February 2019 and December 2019. Each interview was conducted by one of the authors, namely ES or KdG. The interviews were based on an interview guide, including open questions relevant to the objectives of the study (Table 1). The questions in the interview guide were inspired by relevant Dutch legislation [15], the draft of the revised Dutch professional guideline on nursing documentation [19], and the outcomes of a recent survey among nursing staff and focus groups with patients and family caregivers [26].

**Table 1:** Interview guide

1.	In general, do patients participate in nursing documentation in your experience? If not, why not? If so, how?
2.	Do patients understand what is written in electronic health records?
3.	Are there differences between patients in how you let them participate in nursing documentation? If so, which differences do you see?
4.	Under which circumstances do you let patients participate and in which circumstances not?
5.	To what extent is it possible to let patients participate in documentation during all phases of the nursing process? In which phases is it possible, and in which phases is it not possible?
6.	Does the electronic health record that you work with influence the patients participation? If so, how?
7.	If you use an electronic patient portal, what do you gain from using such a portal? And what do you believe patients gain from using an electronic patient portal?
8.	Have you come across challenges in patient participation in nursing documentation? If so, which challenges do you perceive?
9.	How do you deal with the challenges you experience for patient participation in nursing documentation?
10.	How do you think that patient participation in nursing documentation can be made easier for you?
11.	Do you feel that there are differences between the paper-based records and electronic health records regarding patient participation in the nursing documentation?

The interview guide was adjusted after 12 interviews because an interim analysis showed that we had acquired considerable information about experiences regarding patient participation in electronic nursing documentation (objective a), but relatively little information on strategies to address the challenges that nurses face (objective b). For enrichment of the data, we therefore added more in-depth questions to the interview guide regarding strategies for dealing with the challenges nurses encountered.

Seventeen interviews were conducted face-to-face and two by phone. The interviews were scheduled at a place (often the care organization’s office) and time convenient for the participants. All interviews were audio-recorded and transcribed verbatim. The interviews lasted between 18 and 67 min, with an average of 32 min.

### Data analysis

Thematic analysis was performed, using an iterative process of data collection–data analysis–more data collection until data saturation was reached [27]. Within this process, the following six steps of reflexive, inductive thematic analysis were performed: (1) familiarization with the data; (2) generating initial codes; (3) searching for themes; (4) reviewing themes; (5) defining and naming themes; (6) reporting [27].

The transcripts of all 19 interviews were analysed by one author, KdG, and also analysed independently by at least one of the other three authors (ES, WP or AF). All authors had both a nursing background and a scientific background (in nursing science, health science or sociology). The authors compared the codes, themes and interpretations from their analysis; this revealed a high degree of consensus.

### Trustworthiness of the study

The four key criteria of trustworthiness are credibility, generalizability, dependability and confirmability [31]. Credibility concerns the ‘fit’ between participants’ views and the researchers’ representation of those views [31]. One way in which credibility was enhanced was by using triangulation of researchers who interviewed and independently analysed the interview transcripts (see previous section). Another element used to boost credibility was a discussion of the interim and final analysis by the whole team of authors. We also enhanced credibility by carrying out ‘peer debriefing’ with a group of peer researchers who were not involved in the study. The fact that we carried out member checks with the participants also helped the credibility of the study: each participant was presented with a summary of the main themes resulting from the analysis and was invited to give feedback. Member checks were performed after 12 interviews and after data collection had ended. Feedback received from the member checks was discussed within the team of authors. In these discussions the themes were refined until consensus was reached, resulting in the definitive themes.

Another criterion of trustworthiness concerns the generalizability of the inquiry [31]. We have enhanced trustworthiness in this regard by giving descriptions in this article of the setting and the professional backgrounds of the Dutch community nurses (see sections ‘Participants and setting’ and ‘Characteristics of participants’). These descriptions will help those interested in using the findings to judge the transferability of the results to their own situation.

Dependability is another criterion of the trustworthiness of a study. This means that researchers have ensured that the research process is logical, traceable and clearly documented [31]. The dependability of our study is enhanced by the fact that we followed the ‘Standards for reporting qualitative research’ and made sure that the process of coding and analysis was reported in detail [32].

**Fig. 1:**
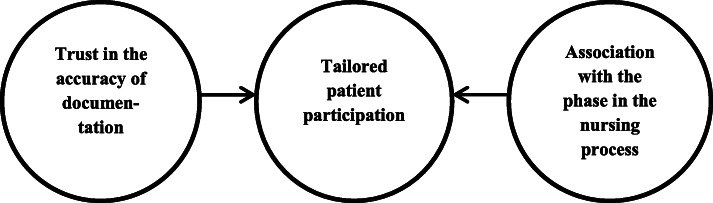
Nurses’ experiences with patient participation in nursing documentation

**Fig. 2:**
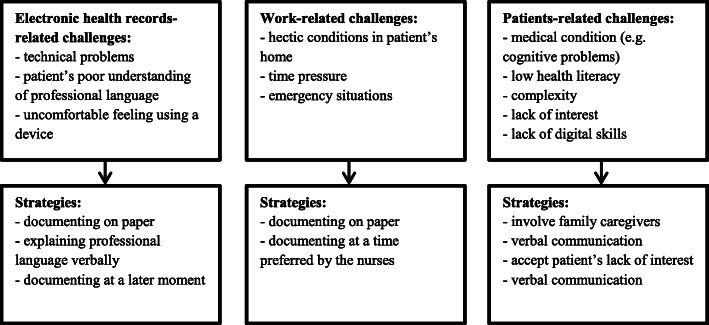
Challenges and strategies regarding patient participation in nursing documentation

Lastly, confirmability is a key criterion of trustworthiness [31]. For confirmability the researcher’s interpretations, findings and conclusions have to be clearly derived from the data. One of the ways we have increased confirmability is by including verbatim statements made by participants in the ‘Results’ section. The fact that we drew various mind maps to visualize the main themes and their interrelatedness also helps the confirmability. The final mind maps are shown in Figs. 1 and 2 of this article.

### Ethical considerations

 The study protocol was approved by the Medical Research Ethics Committee of the Amsterdam University Medical Centre (file number 2019-026). Written informed consent was obtained from all participants. All interviews were audio-recorded, with the approval of the participants. All methods were applied in accordance with relevant guidelines and regulations.

## Results

### Characteristics of participants

Interviews were conducted with 19 participants, 17 of whom were female (Table 2). The participants had between 1 and 39 years of working experience as a registered nurse. Most of the participants worked with the Omaha System (*n* = 16). Participants used electronic health record packages developed by Nedap (*n* = 11), Ecare (*n* = 6) or Unit4 (*n* = 2).

**Table 2:** Characteristics of the participants

Characteristics	N (%)
**Gender**	
Male	2 (10.5)
Female	17 (89.5)
**Educational level**	
Bachelor’s degree	10 (52.6)
Secondary vocational qualification	9 (47.4)
**Age** (years), mean, range	Mean = 35.8, range = 23–56
**Working experience as a nurse**	
0–10 years	13 (68.4)
11–20 years	3 (15.8)
21–30 years	1 (5.3)
31–40 years	2 (10.5)
**Standardized nursing terminology**	
Omaha System	16 (84.2)
NANDA-I NIC NOC	3 (15.8)
**Software developer**	
Nedap	11 (57.9)
Ecare	6 (31.6)
Unit 4	2 (10.5)
**Use of Electronic Patient Portal**	
Yes	18 (94.7)
No	1 (5.3)

### Patient participation in nursing documentation

The interviews revealed three main themes in nurses’ experiences of patient participation in electronic nursing documentation. These are: (1) tailored participation; (2) trust in the accuracy of documentation; (3) association with the phase in the nursing process (Fig. 1).

#### Tailored patient participation

Nurses reported that patient participation in electronic nursing documentation is tailored to the individual situation of patients. For example, in patients with complex care situations (e.g. patients in a terminal stage), nurses often just tell the patient (verbally) what they have documented, while in less complex situations nurses formulated their documentation together with the patient. By telling the patient what they have documented, nurses tried to achieve a passive form of patient participation. Nurses also sometimes deliberately choose not to let very ill patients participate actively in nursing documentation to avoid burdening the patients or giving them more worries.

*“Right, if someone is really sick, I don’t always want to burden them with what I write down or what you hand over to your colleagues. So I don’t always involve them in the documenting then.” (Community nurse 5)*

#### Trust in the accuracy of the documentation

Virtually all nurses found that tailored patient participation is facilitated when patients trust the accuracy of the documentation in electronic health records. Nurses felt that many patients easily trust them to document the right information, just because of their professional relationship. As a result, these patients tend to assume that nursing documentation adequately describes their care needs and the care provided, and therefore they have no wish to participate actively in the documentation process.

*“Then I went and made care agreements with this blind client. So I asked her, ‘How can I leave this behind with you? I mean, I’ve told you everything, but I can’t leave it behind for you to read. […] Then the client said, ‘No, but you work for [organization] so I can assume that whatever’s written there will be the truth.’ […] So she signed the care plan in complete trust.” (Community nurse 6)*

However, some nurses stated that a few patients, often those with a psychiatric condition, have less trust in the accuracy of nurses’ documentation. Because these patients tend to be more suspicious about the accuracy, patient participation in these cases was perceived as challenging by nurses. They differed in how they addressed this challenge. A few nurses engaged in a conversation about the documentation with the patient, while others documented information in less detail.

#### Association with the phase in the nursing process

In addition to trust, nurses stated that tailored patient participation also depends on which phase of the nursing process they are documenting.

Active patient participation, in the sense of formulating documentation together, is limited in the first three phases of the nursing process (i.e. *assessment, nursing diagnosis* and *care planning*). Almost all nurses documented the care needs assessment and drew up the care plan at their office, not in the patient’s home. The nurses interviewed said that this was with good reason, because it takes a lot of time to document the information accurately. After drawing up the care plan, nurses still tried to achieve patient participation by discussing the plan verbally with the patient in their home. Adjustments were then made until the patient fully agreed with the care plan.

*“What I usually do is that I work the assessment out at the office and then give it to the patient and ask them to read it through. Because you’ve just had a long talk with the patient, that’s taken you an hour, and then you can work it out a little more calmly at the office, because otherwise you’re just sitting there typing in front of a patient, and that’s not good when you’re having a talk. It goes quiet. Then I feel you are actually going to miss information.” (Community nurse 18)*

Similarly to the documentation in the first three phases of the nursing process, nurses quite often wrote the progress reports about the *implementation of interventions* in the car or at the office. Nurses often only noted down information on paper in the patient’s home and they then added the information to the electronic health record at a time that was convenient for them. As a result, active participation, in the sense of formulating documentation together, was often limited. Nevertheless, several nurses did say that they discuss the content of the progress reports with patients immediately after giving care and thereby provided an opportunity for active patient participation.

Regarding the *evaluation of care*, the last phase of the nursing process, nurses documented the agreements from the evaluation conversation with patients. Sometimes nurses let patients read the summary in the electronic health records, while in other cases nurses only gave a verbal summary of the agreements made.

All nurses experienced challenges in realizing patient participation during the *handover of care*. Handovers from home care to hospital care in acute situations made patient participation in documentation challenging, if not impossible. The underlying medical condition (e.g. dementia) also made patient participation in documentation challenging in planned handovers, e.g. from home care to nursing homes. Nurses then tried to let patients participate in ways suited to them.

*“You can’t really involve most of the patients who are admitted to nursing homes because of their dementia. So you take a different approach, saying, ‘OK, we’ll make a note so that the nurses there know that you don’t like getting your hair wet in the shower’. But we don’t sit down with them to prepare a handover.” (Community nurse 9)*

### Challenges and strategies in patient participation

Nurses came across various challenges regarding patient participation in nursing documentation (Fig. 2). These challenges were subdivided into three groups, namely those related to electronic health records, work and patients. For each of these groups nurses gave several strategies for dealing with the challenges. Those challenges and strategies are discussed further in the following paragraphs.

#### Electronic health records-related challenges and strategies for them

Some nurses stated that working with electronic health records enabled them to get patients to participate in documentation more often. They noticed that patients were more inclined to give directions about what they wanted to be documented. The nurses believed that using electronic patient portals helped their patients in this respect. However, other nurses found that electronic health records made patient participation in nursing documentation more difficult compared to paper-based health records.

*“Of course, in the past we used the files and you had to write up your reports immediately while at the patient’s home. So at that time, you were forced to do it that way; now you can do it afterwards, but then you haven’t involved your patient in the documenting.” (Community nurse 5)*

Technical problems (e.g. poor internet connections or failures in electronic health records) were noted most frequently by nurses as a challenge for achieving patient participation. These problems often limited nurses’ ability to document information when in the patient’s home, and as result they lost an opportunity to consult patients during documentation. Nurses addressed this challenge by documenting information on paper and adding the information to the electronic health records at a later moment.

In addition to technical problems, two nurses said that the professional language, e.g. derived from the Omaha System or other standardized terminologies, was a challenge for patient participation in documentation. Patients often did not understand certain terms. As a solution, these nurses tried to explain verbally to patients what the terms meant.

*“For example, if you write in the assessment that a patient has pressure ulcers and you’ve started up various actions for that, and the patient kind of feels, ‘OK, but what does this say exactly, this might as well be Greek to me’, then you explain it.” (Community nurse 18)*

Furthermore, more than half of the participants found documenting information on an electronic device (tablet or phone) in the patient’s home to be challenging. It made them feel uncomfortable because the conversation with the patient was interrupted while they typed information into the device. Therefore, some nurses read aloud what they were documenting, while others decided to document information on their tablet or phone at a later moment.

#### Work-related challenges and strategies for them

Nurses often reported that the hectic conditions in the patient’s home, e.g. with the presence of family caregivers or other care professionals, formed a barrier for them to document information there. The same applied to emergency situations, which made patient participation in documentation challenging, if not impossible. Additionally, perceived time pressure frequently prevented nurses from documenting information straight away in the patient’s home.

*“Now we often see colleagues coming to work on their reports at the office because they have a computer there, it’s all a bit bigger, they can do everything at their leisure and they don’t get disturbed. […] When you’re at the patient’s, you have to boot up the tablet, do the report, and the patient either sits there in silence staring into the distance while you’re typing or you get disturbed. And the pressure of, right, I’ve been sitting here for ten minutes now working on my report and I could have already been ten minutes with someone else.” (Community nurse 14)*

#### Patients-related challenges and strategies for them

Nurses felt that the ways for achieving patient participation in electronic nursing documentation were influenced by several patient-related challenges as well.

 Firstly, nurses said the patient’s medical condition played a role in the realization of participation. For instance, they reported that patients with dementia or patients in a terminal stage were barely able to participate in nursing documentation. Nurses then tried to involve family caregivers in the documentation process.

Secondly, nurses felt patient participation was a challenge in situations where the patient had low health literacy. Addressing this challenge, they tried to explain verbally to the patient what information they had documented.

Thirdly, in complex or vulnerable patient situations, e.g. situations with domestic violence, nurses found patient participation more difficult as well. They were highly conscious of what they were writing and took more time to formulate what was being sad, especially when the patient could read what was recorded through an electronic patient portal.

*“That happens when you’re in a situation where there has been maltreatment, for example, or some other form of violence. […] Sometimes things happen that are absolutely not acceptable, and you do need to report what’s going on. I mean, otherwise you have nothing anywhere to refer to later. But you do need to consider how to word it, because a patient might read what you write down. Yet at the same time you need to stay transparent. So it’s a real case of figuring out how you are going to document that.” (Community nurse 10)*

Fourthly, nurses felt that whether patients participated also depended on the individual interest of the patient. Nurses thought that patients often find receiving good nursing care most important, while they attach less importance to participation in documentation. Nurses noted that patients therefore often said that they had no interest in participating. The few patients who nurses remembered as being interested were mostly young, highly educated, or with psychiatric conditions.

In contrast to the limited interest from patients, several nurses had noticed increased interest from family caregivers in nursing documentation since the rise of electronic health records. Family caregivers often read nursing documentation via the electronic patient portal. Even though most nurses were very positive about this trend, others found it somewhat challenging. As a result they were more aware of the phrasing used.

*“We do notice now that the children are far more likely to read it as well, compared with when we had the paper files lying on the table. There really has been a change with the family caregivers being much more active in reading the report and much more active in taking action if there’s anything in the report that draws attention. […] As a result, you start choosing your words even more carefully; you focus more on ‘OK, how should I describe this?’ Because it needs to be clear for everybody, it has to remain respectful and must also still be appropriate for the situation.” (Community nurse 16)*

Lastly, nurses found patient participation to be challenging when patients had limited or no digital skills. Many patients older than about 75 did not have access to a device with an internet connection, let alone know how to use such a device. Nurses believed that participation in electronic nursing documentation is not achievable for many of these patients. However, they did see potential for future generations with more digital skills.

## Discussion

The present study revealed that community nurses felt that patient participation in electronic nursing documentation requires a tailored approach. The extent to which patient participation was realized was influenced by patients’ trust in the accuracy of documentation by nurses, and was associated with the phase of the nursing process that was being documented. Nurses were faced with various challenges relating to electronic health records, the work and the patients. Because of these challenges, nurses often tried to achieve patient participation through verbal communication about what they had documented.

Community nurses considered patient participation in electronic nursing documentation important. This is in line with the current Dutch legislation, which states that patients have the right to supplement, correct and delete information in health records [15].

The finding that patient participation requires a tailored approach is in line with previous studies about patient participation in health care. Patient participation should be tailored to patients’ preferences [33–35]. Active participation is sometimes not preferred by patients as it can be felt to be a burden [33–35]. This corresponds with our finding that community nurses sometimes deliberately choose to let patients participate in nursing documentation passively rather than actively.

Besides a tailored approach, community nurses felt that patient’s trust in the accuracy of the nurses’ documentation is an important aspect facilitating patient participation. The importance of trust between nurses and patients regarding patients’ health information has been underlined in previous research as well [36, 37]. However, a survey study among hospital nurses indicated that electronic health records can put a trusting nurse-patient relationship under pressure [38]. These nurses felt that the computer disrupted their communication with patients [38]. Our study showed similar results, given that community nurses found that doing the documentation in the patient’s home interrupts the conversation with the patient and acts as a barrier.

At the same time, several community nurses in our study stated that patient participation in nursing documentation had improved since the increased use of electronic health records. Electronic patient portals in particular allow patients and family caregivers to read what nurses document and thereby help patients to express what they feel it is important to document. Patients’ input can help to improve the accuracy of nursing documentation, which is of great importance as this accuracy seems to be an issue [5, 9].

A point of particular interest with the use of electronic patient portals, however, is how these portals are arranged. If patient portals are arranged logically according to the phases of the nursing process, they can improve patients’ understanding of nursing documentation and thereby further enhance patient participation in nursing documentation and nursing care. Given that the community nurses felt there were challenges where patients did not understand their documentation, this point deserves some attention.

Arranging the electronic patient portals according to the phases of the nursing process seems to be beneficial, as this provides a logical structure that helps nurses in a methodological approach to working [1, 5]. As a result, such patient portals can improve patients’ understanding of the nurses’ methodological reasoning behind the nursing care provided. Furthermore, if electronic patient portals follow the nursing process, it might also help electronic health records themselves to become better structured according to the same process. Research shows that this is often not the case for the current generation of electronic health records [4, 39].

Moreover, it should be noted that our study and previous research found that many patients older than about 75 lacked the skills required to access and use electronic patient portals [25]. However, community nurses in our study did see potential for patient participation in electronic nursing documentation for the coming generations, who will have better digital skills.

Furthermore, patients’ limited understanding of the professional language used in nursing documentation (e.g. derived from the Omaha System or NANDA-I NIC NOC) was also observed to be a challenge for patient participation. Previous research indicated that written documentation should be supplemented with verbal communication in plain language to ensure patients can understand the information [40]. The nurses who were interviewed also said that written documentation must be combined with verbal communication and explanations.

### Strengths and limitations

A strength of this study is that data saturation was reached, as the last two interviews produced no new aspects that were relevant for our objectives.

A limitation of our study is that we only focused on *nurses’* experiences of patient participation in electronic nursing documentation. As a result, it is not yet known how *patients* perceive their own participation. Future research should focus on gaining a better understanding of patients’ views. Combining these insights with the results of our study will provide a broad perspective on patient participation in electronic nursing documentation. That knowledge will let community nurses and policymakers take action to improve electronic health records and develop efficient strategies for improving patient participation in electronic nursing documentation.

## Conclusions

Community nurses think that patient participation in electronic nursing documentation is important and believe that it requires a tailored approach. Tailored patient participation is facilitated by patients’ trust in the accuracy of the documentation, and associated with the phase of the nursing process that is being documented. Nurses face various challenges relating to electronic health records, the work and the patients (e.g. failures in electronic health records, time pressure and patients’ lack of digital skills). In dealing with these challenges, nurses often fall back on verbal communication with the patient about what was documented in the electronic health records.

## Data Availability

The anonymous transcripts used during the current study are available from the corresponding author on a reasonable request that does not contravene the informed consent forms signed by the participants.
